# Frontiers in Vitamin Research: New Antibodies, New Data

**DOI:** 10.1100/tsw.2011.115

**Published:** 2011-06-09

**Authors:** Rafael Coveñas, Arturo Mangas, Dominique Bodet, Sébastien Duleu, Pilar Marcos, Begum Karakas, Michel Geffard

**Affiliations:** ^1^ Laboratory of Neuroanatomy of the Peptidergic Systems (Lab. 14), Institute of Neurosciences of Castilla y León (INCYL), Salamanca, Spain; ^2^ Department of Physiology and Pharmacology, School of Medicine, University of Salamanca, Salamanca, Spain; ^3^ Immunochemistry Department, GEMAC S.A., Saint Jean d'Illac, France; ^4^ Institut pour le Développement de la Recherche en Pathologie Humaine et Thérapeutique (IDRPHT), Talence, France; ^5^ Laboratory of Human Neuroanatomy, Department of Medical Sciences, School of Medicine, University of Castilla-La Mancha, Albacete, Spain; ^6^ Ege Scientific Research Team (EBAT), School of Medicine, Ege University, Izmir, Turkey; ^7^ IMS Laboratory, ENSCPB-EPHE, Pessac, France

**Keywords:** immunohistochemistry, folic acid, thiamine, riboflavin, vitamin C, pyridoxal, monkey, brain

## Abstract

Since 2004, the anatomical distribution of vitamins in the monkey brain, studied using immunohistochemical techniques and new tools (specific antisera that discriminate different vitamins reasonably well), has been an ongoing research field. The visualization of immunoreactive structures containing vitamins (folic acid, riboflavin, thiamine, pyridoxal, and vitamin C) has recently been reported in the monkey brain (*Macaca fascicularis*), all these vitamins showing a restricted or very restricted distribution. Folic acid, thiamine, and riboflavin have only been observed in immunoreactive fibers, vitamin C has only been found in cell bodies (located in the primary somatosensory cortex), and pyridoxal has been found in both fibers and cell bodies. Perikarya containing pyridoxal have been observed in the paraventricular hypothalamic nucleus, the periventricular hypothalamic region, and in the supraoptic nucleus. The fibers containing vitamins are thick, smooth (without varicosities), and are of medium length or long, whereas immunoreactive cell bodies containing vitamins are round or triangular. At present, there are insufficient data to elucidate the roles played by vitamins in the brain, but the anatomical distribution of these compounds in the monkey brain provides a general idea (although imprecise and requiring much more study) about the possible functional implications of these molecules. In this sense, here the possible functional roles played by vitamins are discussed.

## INTRODUCTION

Vitaminology is an exciting world in which many new lines of research are currently being developed. From the point of view of neuroanatomy, it is a novel avenue of enquiry that is only beginning to be tapped. The complexity of the molecular structures of vitamins and the diversity of their metabolic functions are, at least, impressive and not always easy to understand. Vitamins are widely distributed in nature and many studies have demonstrated the involvement of these molecules in very different metabolic processes[1,2,3]. Vitamins are essential for several reactions since they act as cofactors or coenzymes in many metabolic reactions. Some vitamin deficiencies have been related to certain pathologies and vitamins have been used as preventive medication in different diseases[2,4,5,6,7,8]. In order to detect vitamins in the different organs and fluid samples from several species, biochemical studies using different types of chromatography, vitamin-binding protein assays, enzymatic immunoassays, and radioimmunoassays have been carried out[8,9,10,11,12,13,14,15,16,17,18,19]. However, until now, less knowledge has been garnered about the anatomical localization of these molecules in the mammalian brain.

In recent years, with the advent of highly specific antisera directed against vitamins, the *in situ* detection of these molecules has led to novel studies that have permitted the visualization of immunoreactive structures containing them (e.g., folic acid, riboflavin, thiamine, pyridoxal) in the brain of mammals (in particular, in the monkey brain)[20,21,22,23,24]. The monkey is used in the laboratory as an experimental animal model in order to investigate several scientific issues related to neuroanatomy, neurophysiology, neuropharmacology, and behavior. Results obtained from studies performed in the monkey brain are very important due to its phylogenetical proximity to humans. It is important to examine the distribution of vitamins by immunohistochemistry because this technique provides information about which cells (neurons, glia) display which molecules and where such molecules are located inside the cells (fibers, dendrites, cell body), and because it allows the immunoreactive structures containing vitamins to be located anatomically, not only across a widespread region of the brain (brainstem, thalamus), but also in specific brain nuclei and in layers of the cerebral cortex. Moreover, knowledge about the distribution of immunoreactive structures (fibers and/or cell bodies) containing vitamins in the mammalian brain is necessary in order to gain insight into the role played by these substances in the regions of the brain in which they are present. Thus, in this review, we update the information reported on the anatomical localization of vitamins (folic acid, riboflavin, thiamine, pyridoxal, and vitamin C) in the monkey brain, using new, highly specific antisera against these compounds and immunohistochemical techniques.

## DISTRIBUTION OF FOLIC ACID, RIBOFLAVIN, THIAMINE, PYRIDOXAL, AND VITAMIN C

With single or combined high-performance liquid chromatography (HPLC), the presence of folic acid and/or its derivatives has been carried out in rat bile[25]; liver[26,27]; intestinal mucosae, blood, and the brain[26]; human serum and cerebrospinal fluid[28]; and by folate-binding protein assays in rat liver[29], human placenta[30], choroid plexus, lung, thyroid, and kidney[31]. Moreover, the subcellular distribution of folates has been described in the rat brain[32] and liver[33] using physicochemical methods. In the monkey, the presence of folates has been measured in the liver, kidney, testis, and brain[34], and has been detected using an autoanalyzer in plasma[35].

Riboflavin and/or its derivatives have been detected by HPLC in rat retina[36], brain, heart, liver, and kidney[37]; in rabbit cornea, retina, and blood[38]; in pig liver[39]; and in human blood[10,40,41,42,43]. Moreover, the subcellular distribution (microsomes, mitochondria, plasma membrane) of riboflavin and its coenzyme derivatives has been studied in rat hepatocytes by gel filtration and fluorescence studies of subcellular fractions[44].

Using different HPLC techniques, several studies have addressed the determination of thiamine and/or its derivatives in mouse blood[45] and in the central nervous system of that species[46]; in guinea pig and rat blood[45]; in rat brain[24,47,48], liver, heart, and kidney[47]; in pig liver[39]; in monkey cerebral cortex[49]; and in human blood[45,50,51,52,53], brain[54], and cerebrospinal fluid[50,55]. Moreover, thiamine-binding protein assays have been carried out in the rat digestive tract[19] and brain[18], and microbiological assays have also been carried out in human serum[56]. Finally, the subcellular distribution of thiamine derivatives has also been described in the rat brain[57]. Immunoreactive cells containing thiamine have been reported in the rat cerebellum[24]. In HPLC studies, the presence of thiamine and its derivatives has been described in the monkey cerebral cortex[49] and in the human cerebral cortex, basal ganglia, thalamus, cerebellar cortex, and mammillary bodies[54].

Using HPLC techniques, pyridoxal and/or B6 vitamers have been detected in human plasma/serum/whole blood[10,11,12,13,14] and urine[11,12,14], and in rat plasma, cerebrospinal fluid, and brain[58]. Moreover, pyridoxal bound to human serum albumin has been detected in enzymatic immunoassays[16].

Vitamin C has been detected by HPLC in rat plasma[15] and in pig urinary bladder tissue[17]. In the rat, the presence of vitamin C transporters has been reported in the cerebral cortex and hippocampus using immunocytochemical techniques[59], and the presence of glutathione-dependent dehydroascorbate reductase has been reported in the central nervous system[60]. Moreover, the presence and distribution of vitamin C has been described in the human brain[61] and a mapping of ascorbate distribution has been carried out in the rat brain[62].

Thus, in general, until several years ago, in-depth studies on the distribution of immunoreactive fibers and cell bodies containing vitamins had not been carried out in the mammalian brain. Since 2004, the advent of new, highly specific antibodies directed against folic acid, riboflavin, thiamine, pyridoxal, and vitamin C has allowed us to increase our knowledge of the distribution of vitamins in the mammalian central nervous system and, in particular, in the monkey brain.

## NEW ANTISERA AGAINST VITAMINS

A linkage is the chemical process that combines at least two molecules; thus, depending on the chemical characteristics of the molecules to be linked, the coupling agents used are different[63]. Vitamins (hapten) are small molecules and to be immunogenic, they must be linked to a carrier protein[64,65,66,67]. Thus, a vitamin linked by a coupling agent to the first amino acid of a carrier protein is the antigen target, but the immunogen includes the hapten (e.g., folic acid), the coupling agent, and the whole carrier protein (e.g., bovine serum albumin [BSA]). This means that after immunization, several antibodies will be obtained: antibodies directed against the targeted vitamin (e.g., folic acid) and other antibodies directed against the other components of the immunogen (the carrier protein and coupling agent). Thus, an amalgam of antibodies is obtained. Antibodies directed against the carrier protein and the coupling agent could give a spurious signal that must be blocked, reduced, or suppressed by a later purification and checked after application of the respective controls.

Animals are immunized with one injection every 2 or 3 weeks. In each administration, a mixture of 250 µl of an immunogenic NaCl solution and 250 µl of complete or incomplete Freund adjuvant is injected. The specificity of the antibodies can be improved by changing the carrier protein during the immunization processes (e.g., BSA by human serum albumin). Thus, in the first immunization, the immunogen contains BSA as the carrier protein, whereas in the second immunization, it contains, for example, human serum albumin as the carrier protein instead of BSA. This procedure decreases the amount of antibodies directed against the carrier protein and increases the relative rate of antibodies directed against the targeted molecule (e.g., folic acid)[63]. In a later immunization, the immunogen contains a nonimmunogenic polypeptide, such as Poly-L.Lysine, instead of human/bovine serum albumin. This procedure usually increases the relative amount of antibodies directed against the desired target (e.g., folic acid), improves the affinity and specificity of these antibodies, and decreases the total amount of antibodies directed against the carrier protein and the coupling agent residues. After the second immunization, serum samples are obtained. Prior to performing the enzyme-linked immunosorbent assay (ELISA) and implementing the immunohistochemical technique, the antisera must be prepurified by incubation with the carrier protein–coupling agent in order to remove antibodies directed against these parts of the immunogen, and after the purification process, they must be centrifuged and the floating phase must be purified[63]. Antisera must be tested before and after the purification process in order to evaluate possible alterations that might occur during the purification process, and no changes in the signal or specificity of the antibodies must be found as a consequence of the prepurification process. The antibodies thus obtained must be characterized by ELISA tests. It should be noted that in ELISA tests, the carrier protein–coupling agent must be considered as a competitor in order to prevent possible background noise in later applications. Competitors are selected for the similarity of their chemical structure to that of the target molecule (e.g., folic acid) (see Table 1). The first step in the characterization of the antibodies is to determine the amount of specific antibodies present in the serum; this process is called titration[63]. Once the amount of antibody has been determined, competition experiments are conducted with chemically close molecules and the results of the competition ELISA experiments will show whether the antibody has high or low specificity[20,21,22,23,63,68,69]. For this reason, it is very important to choose adequate competitors. Small differences in chemical structures (e.g., different chain lengths, different chemical groups, and/or different spatial conformations of the same molecule) are good indicators of antibody specificity, since specific antibodies must discriminate among molecules that show a very similar chemical structure to that of the target[70,71,72,73,74]. Thus, several competition experiments are required to confirm the specificity of the antibodies' signal *in vitro* (ELISA) as well as in tissues (immunohistochemical controls). If all the controls are adequate, the signal can be considered specific and not spurious. This was the case of the antivitamin antibodies used and reviewed here[20,21,22,23,63,68,69].

**TABLE 1**
**Affinity and Specificity of Antibodies Directed against Conjugated Folic Acid**

| Competitors | Folic Acid | Cross-reactivity at half-displacement ($IC_{50}$) |
|---|---|---|
| Folic acid–BSA | 1 | |
| Folinic acid–BSA | >1/900 | |
| Tetrahydrofolic acid–BSA | >1/2,000 | |
| Methotrexate–BSA | >1/50,000 | |
| Riboflavin–BSA | >1/50,000 | |
| BSA | >1/50,000 | |

It should be remarked that tissue fixation is of huge importance[20,21,22,23,63,68,69]; not all fixative solutions are suitable for the immunohistochemical visualization of molecules. Thus, for example, picric acid is an effective fixative and is widely used, but it is not appropriate for fixing small molecules, such as vitamins, prior to immunohistochemistry assays due to its slow action[63]. Paraformaldehyde shows good reactivity and fixes small molecules (e.g., vitamins) relatively fast[63]. This latter fixative has been used in studies addressing the distribution of vitamins in the monkey brain[20,21,22,23,68,69]. Incorrect linkage and/or fixation protocols or only partial characterization of the antibodies are generally responsible for the appearance of background in tissues.

Tables 1–5 show the affinity and specificity of antibodies directed against conjugated folic acid–, thiamine–, riboflavin–, pyridoxal–, and vitamin C–BSA. Antibody avidity and specificity were checked using the ELISA method. The estimated antibody avidity ($IC_{50}$) was $10^{-8}$ M for anticonjugated pyridoxal,

**TABLE 2**
**Affinity and Specificity of Antibodies Directed against Conjugated Riboflavin**

| Competitors | Riboflavin | Cross-reactivity at half-displacement ($IC_{50}$) |
|---|---|---|
| Folic acid–BSA | >1/2,600 | |
| Riboflavin–BSA | 1 | |
| BSA | >1/5,000 | |

**TABLE 3**
**Affinity and Specificity of Antibodies Directed against Conjugated Thiamine**

| Competitors | Thiamine | Cross-reactivity at half-displacement ($IC_{50}$) |
|---|---|---|
| Thiamine–BSA | 1 | |
| Folic acid–BSA | >1/80 | |
| Thioctic acid–BSA | >1/50,000 | |
| BSA | >1/50,000 | |

**TABLE 4**
**Affinity and Specificity of Antibodies Directed against Conjugated Pyridoxal**

| Competitors | Pyridoxal | Cross-reactivity at half-displacement ($IC_{50}$) |
|---|---|---|
| Reduced pyridoxal–BSA | 1 | |
| Pyridoxine–BSA | >1/6 | |
| Unreduced pyridoxal–BSA | >1/50,000 | |
| Ascorbic acid–BSA | >1/50,000 | |
| BSA | >1/50,000 | |

**TABLE 5**
**Affinity and Specificity of Antibodies Directed against Conjugated Ascorbic Acid**

| Competitors | Vitamin C | Cross-reactivity at half-displacement ($IC_{50}$) |
|---|---|---|
| Ascorbic acid–BSA | 1 | |
| Dihydroascorbic acid–BSA | 1 | |
| D-Isoascorbic acid–BSA | >1/2,000 | |
| BSA | >1/50,000 | |

folic acid, thiamine, and riboflavin antibodies, and $10^{-7}$ M for anticonjugated vitamin C antibodies[20,21,22,23]. Moreover, the specificity of these antibodies was very high, since, for example, the anti–folic acid antibody discriminated conjugated folic acid from other conjugated molecules (methotrexate, folinic acid, tetrahydrofolic acid, riboflavin, and BSA) very well (Table 1).

## ANATOMICAL DISTRIBUTION OF VITAMINS IN THE MONKEY BRAIN: NEW ANTIBODIES, NEW DATA

Polyclonal primary antisera against folic acid, riboflavin, thiamine, pyridoxal, and vitamin C were obtained from commercial sources (GEMAC S.A., Saint Jean d'Illac, France) and were raised in rats or rabbits with their respective BSA immunogens. In this sense, antibodies directed against folic acid, thiamine, riboflavin, and vitamin C were developed in rats[20,21,22,23], whereas antibodies directed against pyridoxal were developed in rabbits[63]. Tables 6 and 7 show the distribution of vitamins in the monkey (*Macaca fascicularis*) brain according to new, highly specific antibodies and immunohistochemical techniques[20,21,22,23,68,69]. It should be noted that the immunoreactive structures containing the vitamins show a very restricted distribution in the monkey brain (Figs. 1–3). Folic acid (vitamin $B_9$) showed the most widespread distribution (immunoreactive fibers were found in 13 of the 19 nuclei/regions in which immunoreactivity for vitamins has been observed in that species) (Figs. 1–3, 4A,B), followed by thiamine (vitamin $B_1$) (9/19) (Figs. 1–3, 4C), riboflavin (vitamin $B_2$) (7/19) (Figs. 1–3, 4D), pyridoxal (the natural form of vitamin $B_6$) (4/19) (Figs. 3A,C, 4E,F), and vitamin C (ascorbic acid) (1/19) (Figs. 1A, 4G). Vitamin C was only detected in the primary somatosensory cortex and, to date, no other vitamin has been reported in this region (Figs. 1A, 4G). Folic acid, thiamine, and riboflavin were only observed in immunoreactive fibers (Figs. 1–3, 4A–D), whereas vitamin C was only found in cell bodies. However, pyridoxal was found in both fibers and cell bodies. Perikarya containing pyridoxal were located in the paraventricular hypothalamic nucleus (Figs. 3A, 4E), the periventricular hypothalamic region (Fig. 3A), and in the supraoptic nucleus (Figs. 3A, 4F). Another two vitamins were studied in the monkey brain — pyridoxine (another natural form of vitamin $B_6$) and nicotinamide (the amide of nicotinic acid or vitamin $B_3$) — but no immunoreactive structure containing them was observed[20]. None of the five vitamins were found together in the nuclei/regions described in Tables 6 and 7, due to the fact that the distribution of vitamin C and pyridoxal is quite different from the distribution of folic acid, thiamine, and riboflavin. Regarding these latter three vitamins, some of the anatomical localizations where these vitamins were observed are common (e.g., the pulvinar nucleus, the lateral posterior nucleus, the region extending from the pulvinar nucleus to the caudate nucleus, and the medial geniculate nucleus) (Fig. 4C,D), but in other anatomical regions, only one of these vitamins (folic acid or thiamine) was present in the immunoreactive fibers (Table 6). In this sense, in six of the 13 nuclei/regions in which immunoreactive fibers containing folic acid were found, riboflavin-immunoreactive fibers were also observed; thiamine-immunoreactive fibers were also found in six of them. Currently, whether the three mentioned vitamins coexist or not in the same fibers remains unknown.

It is remarkable to note that, in general, the density of the immunoreactive fibers containing vitamins was low, whereas in a few areas, only moderate or high levels of such fibers were found (e.g., fibers containing thiamine located in the region extending from the pulvinar nucleus to the caudate nucleus) (Table 6). Moreover, in many nuclei of the brain monkey, single fibers containing vitamins were found (Table 6). For the time being, the morphological characteristics of the immunoreactive fibers containing vitamins in the monkey brain are identical; they were thick, smooth (without varicosities), and of medium length or long (Fig. 4A–D). In general, the immunoreactive cell bodies containing pyridoxal or vitamin C were round or triangular (Fig. 4E–G). The vast majority of these immunoreactive cell bodies was small, and showed none or one to two short visible processes (Fig. 4E–G). It should also be remarked that after the application of a standard immunohistochemical technique or an immunohistochemical method that enhances the immunohistochemical reaction product[20,68], the same pattern of distribution for vitamin-immunoreactive cell bodies in the brain was observed, with respect to that found when the enhancing method

**TABLE 6**
**Neuroanatomical Distribution of Folic Acid, Thiamine, and Riboflavin in the Monkey Brain (*Macaca fascicularis*)**

| Anatomical Structure | Folic Acid | Thiamine | Riboflavin |
|---|---|---|---|
| Anterior commissure | — | F (+) | F (+) |
| Centrum medianum-parafascicular complex | F (s) | F (s) | — |
| Cerebral cortex | — | F (+) | — |
| Dorsomedial nucleus | F (s) | — | — |
| Inferior pulvinar nucleus | F (s) | F (+) | — |
| Lateral geniculate nucleus | F (s) | — | — |
| Lateral posterior nucleus | F (s) | F (+) | F (s) |
| Medial geniculate nucleus | F (s) | F (+) | F (s) |
| Medial lemniscus | — | F (s) | — |
| Pulvinar nucleus | F (++) | F (++) | F (+++) |
| Region extending from the pulvinar nucleus to the caudate nucleus | F (s) | F(++) | F (+++) |
| Region extending from the zone above the substantia nigra to the habenular complex | F (+) | — | — |
| Region located above the medial and lateral geniculate nuclei | F (+) | — | F (+) |
| Region located above the substantia nigra | F (+) | — | F (+++) |
| Ventral posteroinferior nucleus | F (s) | — | — |
| Ventral posterormedial nucleus, parvocellular part | F (s) | — | — |

F (fibers): s (single); + (low density); ++ (moderate density); +++ (high density).

**TABLE 7**
**Neuroanatomical Distribution of Pyridoxal and Vitamin C in the Monkey Brain (*Macaca fascicularis*)**

| Anatomical Structure | Pyridoxal | Vitamin C |
|---|---|---|
| Anterior commissure | F (+) | — |
| Cerebral cortex | — | CB (++) |
| Periventricular hypothalamic region | CB (+) | — |
| Paraventricular hypothalamic nucleus | CB (+++) | — |
| Supraoptic nucleus | CB (+++) | — |

CB (cell bodies): + (low density: <10 cell bodies/region/section); ++ (moderate density: 10–20 cell bodies/region/section); +++ (high density: >20 cell bodies/region/section). F (fibers): + (low density).

was not used. Moreover, following the use of both methods, the number of immunoreactive cell bodies found was fairly similar. However, the degree of immunoreactivity was higher when the enhancing method was used and in some cases using this method, long dendrites were observed, although they were not found when the nonenhancing method was applied[20].

In immunohistochemical studies, in order to discard spurious signals, it is very important to carry out the pertinent controls to confirm the specificity of the immunoreactivity. The tests generally used to show the specificity of the immunoreactivity observed in nerve tissue are not absolute and the validity of the results should always be checked with other techniques. In this sense, the affinity and specificity of antibodies

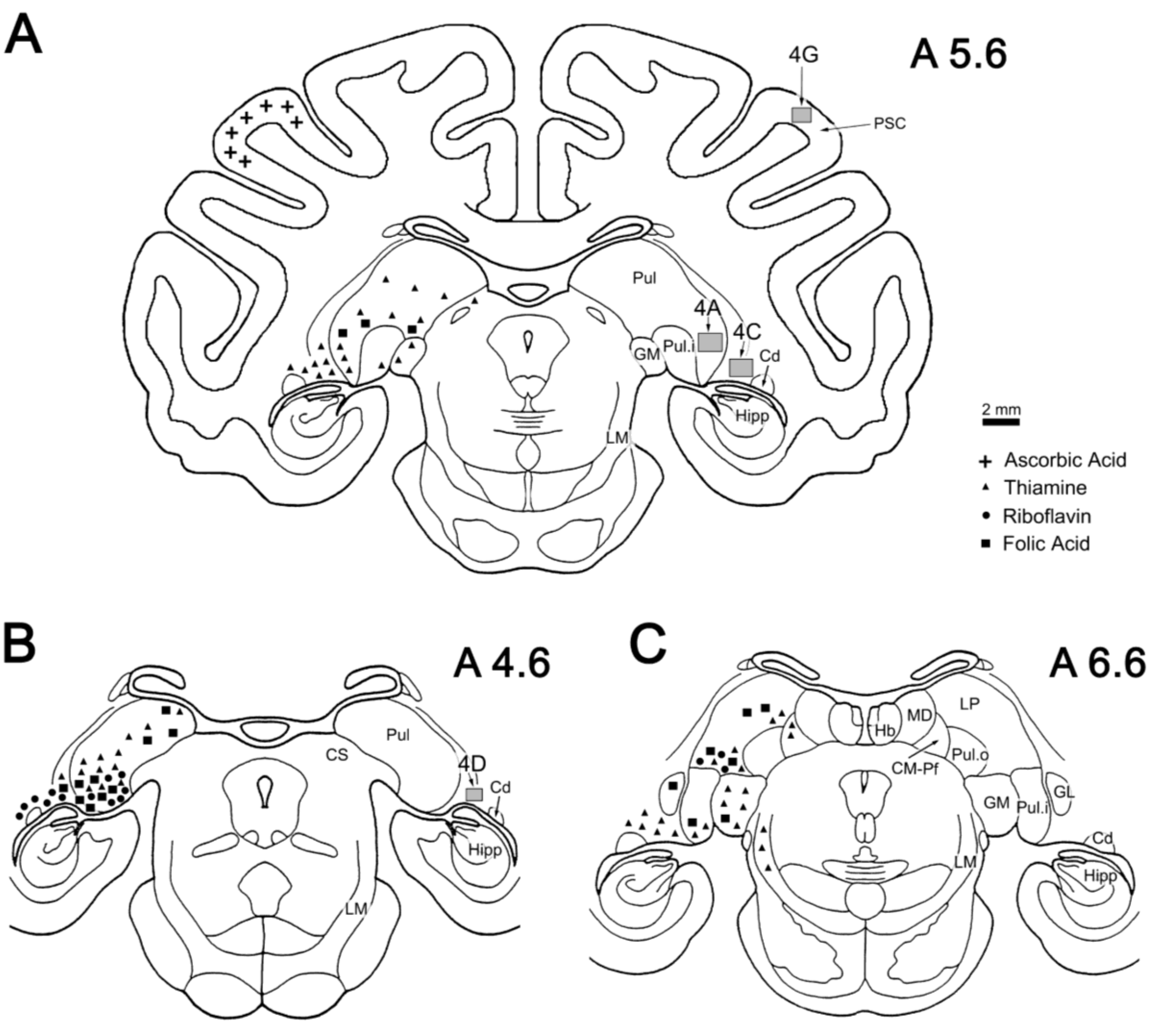

**FIGURE 1.** Distribution of ascorbic acid (cell bodies), thiamine (fibers), riboflavin (fibers), and folic acid (fibers) in frontal planes of the monkey (*Macaca fascicularis*) brain according to the stereotaxic atlas of Szabo and Cowan[95]. In each section, the anteriority (A) in millimeters with respect to the zero stereotaxic point is indicated at the upper right. Abbreviations: Cd: caudate nucleus; CM-Pf: centrum medianum-parafascicular complex; CS: superior colliculus; GL: lateral geniculate nucleus; GM: medial geniculate nucleus; Hb: habenula complex; Hipp: hippocampus; LM: medial lemniscus; LP: lateral posterior nucleus; MD: dorsomedial nucleus; PSC: primary somatosensory cortex; Pul: pulvinar; Pul.i: inferior pulvinar nucleus; Pul.o: oral pulvinar nucleus.

directed against vitamins must be tested using ELISA tests and they must also be studied under the same conditions with different competitors in each particular case. Moreover, classical immunohistochemical controls (omission of primary and secondary antibodies; incubation of the primary antibody with their specific targets linked to the protein carrier [e.g., anticonjugated vitamin C antibodies with conjugated vitamin C–BSA]) must be carried out in order to confirm that the antibodies are indeed specific against the vitamins. In sum, as a general rule, the immunohistochemical results obtained in nerve tissue must also be confirmed by the results from the ELISA techniques. This is the case for all the antibodies used against the vitamins studied in the monkey brain[20,21,22,23].

The data reported above suggest that vitamins have different anatomical distributions in the monkey brain and that such distributions are very restricted. However, the possibility that the immunohistochemical techniques used might be insufficiently sensitive to visualize all the profiles containing vitamins in the brain

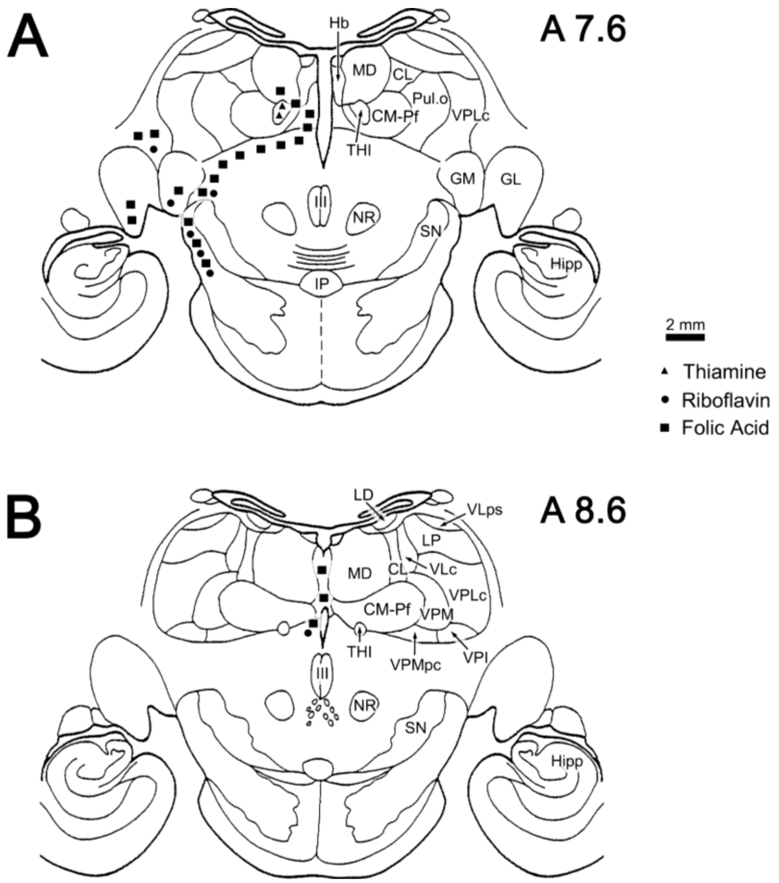

**FIGURE 2.** Distribution of thiamine-, riboflavin-, and folic acid–immunoreactive fibers in frontal planes of the monkey (*Macaca fascicularis*) brain according to the stereotaxic atlas of Szabo and Cowan[95]. In each section, the anteriority (A) in millimeters with respect to the zero stereotaxic point is indicated at the upper right. Abbreviations: III: oculomotor nucleus; CL: central lateral nucleus; CM-Pf: centrum medianum-parafascicular complex; GL: lateral geniculate nucleus; GM: medial geniculate nucleus; Hb: habenula complex; Hipp : hippocampus; IP: interpeduncular nucleus; LD: lateral dorsal nucleus; LP : lateral posterior nucleus; MD: dorsomedial nucleus; NR: red nucleus; Pul.o: oral pulvinar nucleus; SN: substantia nigra; THI: habenulo-interpeduncular tract; VLc: ventral lateral nucleus, caudal part; VLps: ventral lateral nucleus, pars postrema; VPI: ventral posteroinferior nucleus; VPLc: ventral posterolateral nucleus, caudal part; VPM: ventral posteromedial nucleus; VPMpc: ventral posteromedial nucleus, parvocellular part.

cannot be ruled out. Thus, it is possible that other neuronal structures might contain low levels of vitamins, undetectable with the technical approach used.

## POSSIBLE FUNCTIONAL ROLES OF VITAMINS

The involvement of vitamins in metabolic processes is well known, although their neuroanatomical distributions suggest that they may play hitherto unsuspected roles. Thus, the roles of vitamins could vary depending on the nuclei of the brain in which they are located.

According to the distribution of folic acid in the monkey brain, this vitamin could be involved in auditory, visual, motor, and somatosensorial mechanisms. Folic acid has been involved in several intracellular reactions as a cofactor, and the reduced derivatives of folic acid are essential coenzymes for the biosynthesis of purine nucleotides, methionine, and thymidylate, and are also involved in reactions of transfer, oxidation, and the reduction of single carbon units[75]. It has also been reported that higher folic acid intake may reduce the risk of vascular disease and prevent neural tube defects, and that an intake of folate could reduce the risk of cancer[76,77].

One of the most important roles of riboflavin is as a precursor of coenzymes such as flavin mononucleotide (FMN) and flavin adenine dinucleotide (FAD), both involved in several metabolic processes. Riboflavin has been located in the pulvinar nucleus, suggesting that it could be involved in visual mechanisms. Moreover, in this thalamic nucleus, a reduction in volume has been reported in patients with schizophrenia or with schizotypal personality disorder[78,79]. In the future, a possible correlation between the level of riboflavin in the pulvinar nucleus and pathological implications in schizophrenia should be investigated in depth (e.g., regulating cell volume and/or cell death).

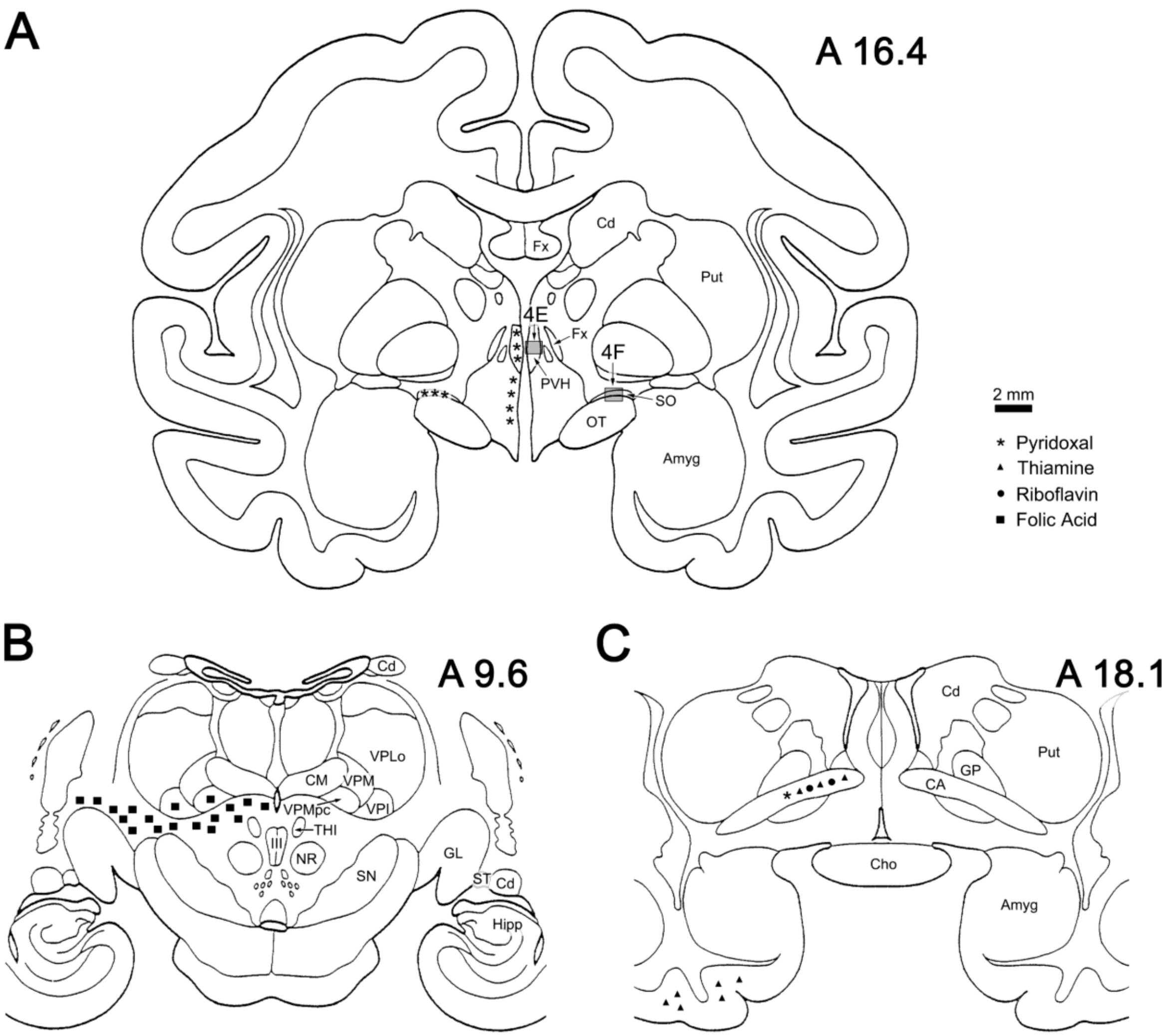

**FIGURE 3.** Distribution of thiamine-, riboflavin-, and folic acid–immunoreactive fibers and pyridoxal-immunoreactive cell bodies (A 16.4) and fibers (A 18.1) in frontal planes of the monkey (*Macaca fascicularis*) brain according to the stereotaxic atlas of Szabo and Cowan[95]. In each section, the anteriority (A) in millimeters with respect to the zero stereotaxic point is indicated at the upper right. Abbreviations: III: oculomotor nucleus; Amyg: amygdala; CA: anterior commissure; Cd: caudate nucleus; Cho: optic chiasma; CM: centrum medianum; Fx: fornix; GL: lateral geniculate nucleus; GP: globus pallidus; Hipp : hippocampus; NR: red nucleus; OT: optic tract; Put: putamen; PVH: paraventricular hypothalamic nucleus; SN: substantia nigra; SO: supraoptic nucleus; ST: stria terminalis; THI: habenulo-interpeduncular tract; VPI: ventral posteroinferior nucleus; VPLo: ventral posterolateral nucleus, oral part; VPM: ventral posteromedial nucleus; VPMpc: ventral posteromedial nucleus, parvocellular part.

Thiamine has been implicated in the synthesis of acetylcholine, GABA, glutamate, and aspartate[80]. It has also been reported that this vitamin affects membrane ion channels, probably involving potassium channels[81], and that thiamine exerts antioxidant and neurotrophic actions[82]. This neurotrophic action could be due to its coenzymatic role in biochemical reactions and/or its specific function in neurotransmission and nerve conduction[82]. Moreover, it is known that thiamine is a coenzyme involved in the decarboxylation of α ketoacids and in transketolation, that it plays an essential role in carbohydrate metabolism, and that a change in thiamine status is associated with certain clinical states, such as the Wernicke-Korsakoff syndrome and beriberi[83]. Moreover, thiamine deficiency has been implicated in

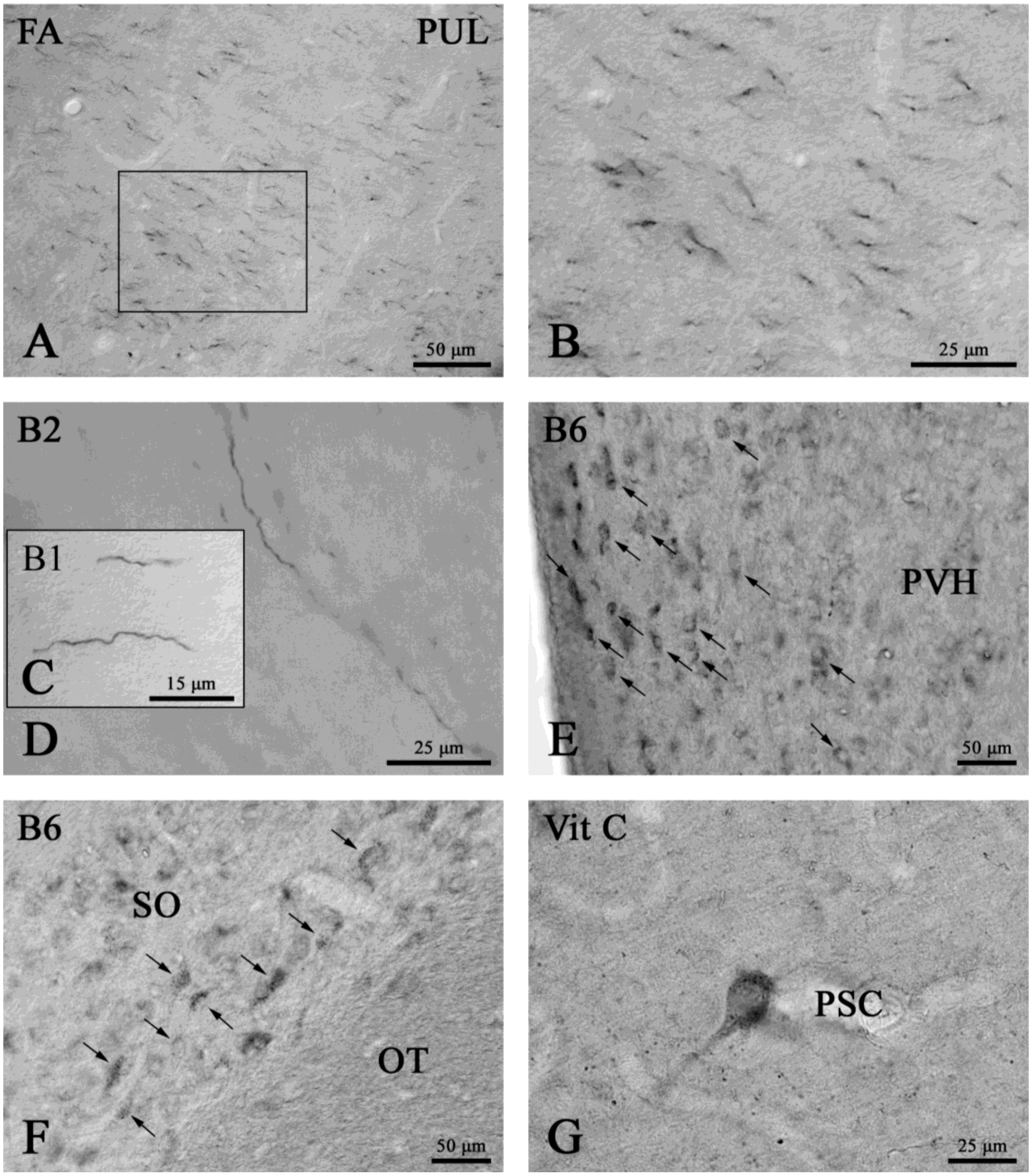

**FIGURE 4.** Immunoreactive fibers and cell bodies containing vitamins in the monkey brain. (A) Immunoreactive fibers containing folic acid (FA) located in the pulvinar nucleus (PUL). (B) A high-power magnification of the region delimited in Fig. 4A. Photographs A and B were taken from the region indicated as 4A in Fig. 1A. (C) Thiamine (B1)-immunoreactive fibers in the region extending from the pulvinar nucleus to the caudate nucleus. This photograph was taken from the region indicated as 4C in Fig. 1A. (D) Immunoreactive fibers containing riboflavin (B2) located in the region extending from the pulvinar nucleus to the caudate nucleus. This photograph was taken from the region indicated as 4D in Fig. 1B. (E) Cell bodies (arrows) containing pyridoxal (B6) located in the paraventricular hypothalamic nucleus (PVH). This photograph was taken from the region indicated as 4E in Fig. 3A. (F) Pyridoxal (B6)-immunoreactive cell bodies (arrows) located in the supraoptic nucleus (SO). OT: optic tract. This photograph was taken from the region indicated as 4F in Fig. 3A. (G) A high-power magnification of an immunoreactive cell body containing vitamin C (Vit C) located in the primary somatosensory cortex (PSC). This photograph was taken from the region indicated as 4G in Fig. 1A. Fig. 4D is published with permission of GEMAC S.A. Laboratories.

mechanisms of selective neuronal cell death[84]. According to the anatomical distribution reported for thiamine in the monkey brain, this vitamin could be involved in auditory and visual mechanisms, since immunoreactive fibers containing thiamine have been located in the medial geniculate nucleus and in the pulvinar nucleus.

The presence of pyridoxal in the paraventricular hypothalamic nucleus suggests that this vitamin could be involved in stress, food intake, and neuroendocrine mechanisms[85,86]. The coexistence of pyridoxal with vasopressin, oxytocin, or corticotropin-releasing hormone, as well as the presence of this vitamin in AIDS or Parkinson’s disease patients, should be investigated in the future since in the paraventricular hypothalamic nucleus, a decrease in the number of neurons containing oxytocin has been reported in both diseases[86]. Moreover, due to its antioxidant properties, pyridoxal has been related to the prevention of several disorders.

Vitamin C has been proposed to be a neuromodulator of both dopamine- and glutamate-mediated neurotransmission, and it is an essential cofactor for noradrenaline synthesis. It is also required for the release of noradrenaline and acetylcholine from synaptic vesicles, and it acts as a cofactor in the synthesis of many neuropeptides[1,87]. It has also been reported that the content of ascorbate in the white matter is much lower than that found in the gray matter[87]. In the monkey, vitamin C–positive cortical neurons have been located only in the primary somatosensory cortex. In the human cortex, scarce fibers containing vitamin C have been found[61]. It has also been described that the cortical content of vitamin C in mammals decreases phylogenetically; it is higher in the mouse than in rats, and higher in these species than in humans[1]. Physiologically, vitamin C is present as the ascorbate anion. Humans and nonhuman primates cannot synthesize ascorbate from glucose, unlike other animals that have the enzyme required for the last step in ascorbate biosynthesis. Thus, ascorbate is absorbed from the diet and enters the central nervous system[1]. Ascorbate levels are modulated by a glutamate–ascorbate heteroexchange across cell membranes, mainly in neurons[88]. The presence of vitamin C in monkey cortical neurons could be due to this heteroexchange process. This mechanism might provide neuroprotection by facilitating glutamate uptake or by increasing ascorbate concentrations, thus minimizing the excitotoxic consequences of glutamate release. Vitamin C is also able to decrease lipid peroxidation levels[89], exerting a potent neuroprotective effect. Moreover, ascorbic acid is a component of a new drug candidate (GEMSP) tested in the treatment of multiple sclerosis owing to its interactions with reactive oxygen species (ROS) and nitric oxide(NO)[90].

In addition, many studies have shown the therapeutic effects of vitamins. Thus, (1) vitamin K has been used as a possible therapy in an animal model of multiple sclerosis[91], (2) populations with a regular intake of vitamin E have a lower risk of amyotrophic lateral sclerosis than nonuser populations[92], (3) α-tocopherol succinate is a component of GEMSP owing to its interactions with ROS and NO[93], and (4) a vitamin A metabolite (all-trans retinoic acid) has been shown to exert a therapeutic effect in an experimental autoimmune nephritis model[94]. The possible therapeutic implications of vitamins open new perspectives as regards the involvement of such substances in neurodegenerative diseases. In this sense, in the future, the relationship between the absence or low levels of vitamins and the development of neurodegenerative disorders should be studied. If this relationship exists, vitamins could play a more important role than merely exerting a purely metabolic action and studies on the distribution of vitamin-immunoreactive structures in the mammalian brain, and in particular in primates, would be of huge importance.

## FUTURE STUDIES ON VITAMINS

Future experiments should be carried out in order to collect more data and gain further insight into the involvement of vitamins in the monkey brain. Thus, such studies should attempt to unravel whether these molecules act as neuromodulators/neurotransmitters, or whether an uptake of these vitamins occurs, as has been demonstrated for other substances (D-glutamate)[70]. Moreover, it would be particularly interesting to study the regions/nuclei of the monkey brain in which two or more vitamins have been localized in the same places in order to determine the functional relationships among them. Future studies should also focus on the anatomical distribution of more vitamins in the monkey brain. In this sense, new antibodies directed against other vitamins (e.g., pantothenic acid, α-tocopherol, retinoic acid) other than those reported here should be developed. Moreover, the origin of the immunoreactive fibers containing vitamins in the monkey brain is unknown. Thus, future studies should attempt to demonstrate the localization of the cell bodies from which the immunoreactive fibers containing vitamins arise. Finally, it would be very interesting to study the distribution of vitamins in the human brain using new, highly specific antisera against them.

It should be remarked that it is not always possible to test the specificity of antibodies with close molecules/structures/vitamers. Thus, for example, the specificity of antibodies directed against thiamine were not evaluated either with thiamine monophosphate or with thiamine diphosphate[22]. Likewise, different vitamers of the same vitamin (e.g., $B_6$ vitamin) or *in vivo* active molecules (e.g., thiamine diphosphate) are not always tested. This is because of the impossibility of binding these molecules in a stable way, at least for the time being. Accordingly, it is of huge importance to continue developing new linkages that will allow these molecules, currently unavailable, to be tested.

In sum, in order to better determine the distribution and roles of vitamins in the mammalian brain, new antibodies should be directed against these molecules and it is also necessary to search for different coupling methods that could improve the results obtained with currently available techniques. By using immunohistochemical techniques, vitamins could be readily visualized and such techniques may be considered suitable tools for improving our knowledge about the distribution and function of vitamins in different tissues, especially in the central nervous systems of different species. At present, the specific and special functional role that vitamins might play in the monkey brain is unknown. In this sense, additional neuroanatomical and physiological studies should be carried out in order to shed further light on the functional role of vitamins in the monkey brain.

## ACKNOWLEDGMENTS

This work has been supported by GEMAC S.A. Laboratories (Saint Jean d'Illac, France) and IDRPHT (Talence, France). The authors wish to thank Mr. G. Glaize for technical assistance and Mr. N. Skinner for supervising the English text.

## CONFLICT OF INTEREST STATEMENT

D. Bodet and M. Geffard are ascribed to the company GEMAC S.A. which partially supported the work. This company was the source of the antibodies used in the studies reviewed.
